# Pitting Initiation and Propagation of X70 Pipeline Steel Exposed to Chloride-Containing Environments

**DOI:** 10.3390/ma10091076

**Published:** 2017-09-13

**Authors:** Zixuan Yang, Bo Kan, Jinxu Li, Yanjing Su, Lijie Qiao, Alex A. Volinsky

**Affiliations:** 1 Corrosion and Protection Center, Key Laboratory for Environmental Fracture (MOE), University of Science and Technology Beijing, Beijing 100083, China; Zixuan_Yang2017@163.com (Z.Y.); Kanbo10008@163.com (B.K.); yjsu@ustb.edu.cn (Y.S.); lqiao@ustb.edu.cn (L.Q.); 2 Department of Mechanical Engineering, University of South Florida, Tampa, FL 33620, USA; volinsky@usf.edu

**Keywords:** pitting corrosion, inclusions, SKPFM, X70 pipeline steel

## Abstract

Inclusion-induced pitting initiation mechanisms in X70 steel were investigated by scanning electron microscopy, scanning Kelvin probe force microscopy (SKPFM), immersion and electrochemical polarization tests in chloride-containing ion solutions. There are three inclusion types in the X70 steel. Corrosion test results indicated that pitting corrosion resistance of type A inclusion < type C inclusion < type B inclusion, i.e., (Mn, Ca)S < matrix < (Al, Ca)O. SKPFM test results show that the type A inclusion exhibited both lower and higher potentials than the matrix, while the type B inclusion exhibited higher potential than the matrix. The corrosion test and the SKPFM potential test results are consistent. Potentiodynamic polarization results indicate that the type A and C are active inclusions, while the type B is an inactive inclusion. Three kinds of possible mechanisms of inclusion-induced pitting corrosion are established for the X70 steel.

## 1. Introduction

Pitting corrosion is the primary origin of steel failure in corrosive environments. Pitting generally occurs in a small area of metal surface and causes devices to fail by perforation or initiates stress corrosion cracks [1,2]. Thus, it is necessary to understand pitting corrosion mechanisms in steel.

Previous researchers have reported pitting initiation being caused by localized electrodissolution of metal at inclusions [3,4]. They found that carbon steel and stainless steel have several different kinds of inclusions, such as MnS, Al_2_O_3_, CaO, AlN, along with some complex inclusions [5,6,7]. These inclusions are quite different in different steels. Thus, many contested models of pitting corrosion exist [8,9,10,11,12,13,14,15,16]. In addition, the prediction of pitting events remains very difficult. Williams et al. proposed that chemical changes in and around MnS inclusions are responsible for pitting initiation [8]. Zheng et al. reported that the nano MnCr_2_O_4_ in MnS inclusions can promote pitting initiation [11]. Punket et al. found that some pits are active and others are inactive. However, they did not show what types of inclusions are active [13]. Lin et al. studied initiation of pitting corrosion in carbon steel and found that some metastable pitting nuclei developed into macro-corrosion pits but others lost their corrosion activity [16]. Thus, the pitting process has been described as random and sporadic by previous researchers [17].

However, one undisputed aspect is that inclusions play a critical role in inducing pitting [18,19,20]. Previous research focused on studying the effects of single MnS inclusions in stainless steel [21,22]. It is now known that the inclusions in the X70 steel are not single, but composite inclusions. Chemical and structural changes in inclusions determine the pitting initiation mechanisms. Usually, pipeline steel is used for oil transport in soil or marine environments. Inevitably, chloride ions exist in the working environment. Thus, we illustrate the mechanisms of pitting corrosion for three kinds of inclusions in the X70 pipeline steel in chloride-containing environments. In addition, the pitting resistance of the three kinds of inclusions are compared, which is also valuable for the X70 steel smelting.

For these purposes, the corrosion process of the X70 steel was examined in different chloride-containing environments in this study. The scanning Kelvin probe force microscopy (SKPFM), scanning electron microscopy (SEM) and energy dispersive spectroscopy (EDS) were utilized to investigate the potential, structure and chemical composition of inclusions in the X70 steel.

## 2. Experimental Procedure

### 2.1. Materials and Samples Preparation

X70 pipeline steel was used for experiments with the chemical composition listed in Table 1. Specimens were not heat treated before the tests. Small specimens for potentiostatic and potentiodynamic polarization tests with 2 mm × 2 mm × 5 mm size were cut from the X70 plates. The non-working surface of the specimen was sealed with silicone. Standard corrosion specimens (10 mm × 10 mm × 5 mm) were used for corrosion tests and square specimens with the 10 mm side for the SKPFM measurements. The working face of the specimens was sanded with emery paper from 400 to 5000 number and then polished with 1 μm diamond paste to avoid surface roughness effects on pitting corrosion. Finally, the specimens were ultrasonically cleaned in ethanol.

### 2.2. Microscopy Observations

In order to understand the effect of surface inclusions on pitting corrosion nucleation, the composition and structure of the inclusions in the X70 plate were observed by SEM (Zeiss, EVO MA 10/LS 10, Oberkochen, Germany) equipped with energy dispersive spectroscopy (EDS). The inclusions’ morphology was obtained by SEM and the composition was detected by EDS mapping. The surface morphology and contact potential difference (CPD) around the inclusions in the X70 steel were obtained using atomic force microscopy (AFM, Agilent, 5500 AFM/SPM, Santa Clara, CA, USA). Scanning Kelvin probe force microscopy (SKPFM, Agilent, 5500 AFM/SPM, Santa Clara, CA, USA) was performed on polished samples. Before the SKPFM tests, the samples were marked using a micro-hardness tester. The contact potential around inclusions was obtained from the line scan analysis of 512 × 512 images acquired at 1 Hz.

### 2.3. Corrosion Tests 

Corrosion tests were conducted to analyze the pitting corrosion resistance of the X70 steel in a simulated chlorine deposit environment. The corrosion environment was simulated using 0.1 mol/L NaCl + 0.5 mol/L NaHCO_3_ and 0.5 mol/L NaCl neutral pH solutions. Deionized water and analytical grade chemicals were used to prepare the solutions. Prior to the experiments, the solutions were purged of air with nitrogen. The surfaces of the corroded steel specimens in each environment after different immersion times were examined by SEM and EDS. The inclusions and metastable pits were observed by SEM again to detect the dissolution morphology and composition.

### 2.4. Electrochemical Measurements

Electrochemical measurements were carried out in 0.5 mol/L NaHCO_3_ + 0.1 mol/L NaCl neutral pH solution using an electrochemical workstation (Gamry, Reference 6000, Warminster, PA, USA). The solution was prepared using analytical grade chemicals and deionized water. This solution was used because it is gentler than the 3.5% NaCl solution to investigate the corrosion process. The electrochemical experimental device was comprised of the three electrodes system. The counter electrode was the platinum foil and the reference electrode was the Ag/AgCl electrode. All electrochemical evaluations were performed at 25 °C and all potentials quoted in this work refer to the Ag/AgCl electrode.

During potentiodynamic polarization measurements the potential was scanned from 100 mV below the open circuit potential. The potential scanning rate was kept at 0.2 mV/s in anodic direction until stable pitting happened. The data acquisition rate was 20 Hz and no data smoothing was applied. The morphology of the inclusions at different potentials was observed by SEM. Each electrochemical measurement was repeated three times under the same conditions.

## 3. Results and Discussion

### 3.1. Corrosion Tests

**Figure 1:**
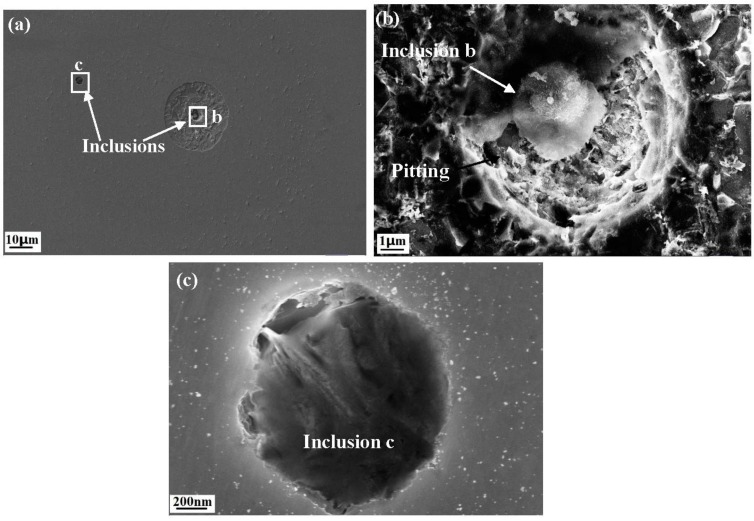
Corrosion morphology of the X70 steel after 5 min immersion in 0.5 mol/L NaCl solution: (**a**) overall morphology; local morphology of (**b**) inclusion b and (**c**) inclusion c.

Figure 1 shows the X70 steel surface after 5 min immersion in 0.5 mol/L NaCl solution at 25 °C. There are two inclusions in the image, one of which had induced pitting, but the other did not. Figure 1a shows that corrosion initiates from the inclusion site and the corrosion products precipitate around the inclusion. Two inclusions are enlarged in Figure 1b,c. The elemental percentages of the two types of inclusions in Figure 1, obtained by EDS, are listed in Table 2. Based on the chemical composition, inclusions at the b position contain more sulphide, although it had been partially dissolved. These results indicate that different inclusions have different pitting resistance. The content of sulphide in inclusions plays a critical role in pitting initiation. Inclusions have both active and non-active components, and an active inclusion is an anode, which gets dissolved first. These results show good agreement with the pitting behavior of inclusions in steel [11,13].

### 3.2. SEM Inclusions Observations

**Figure 2:**
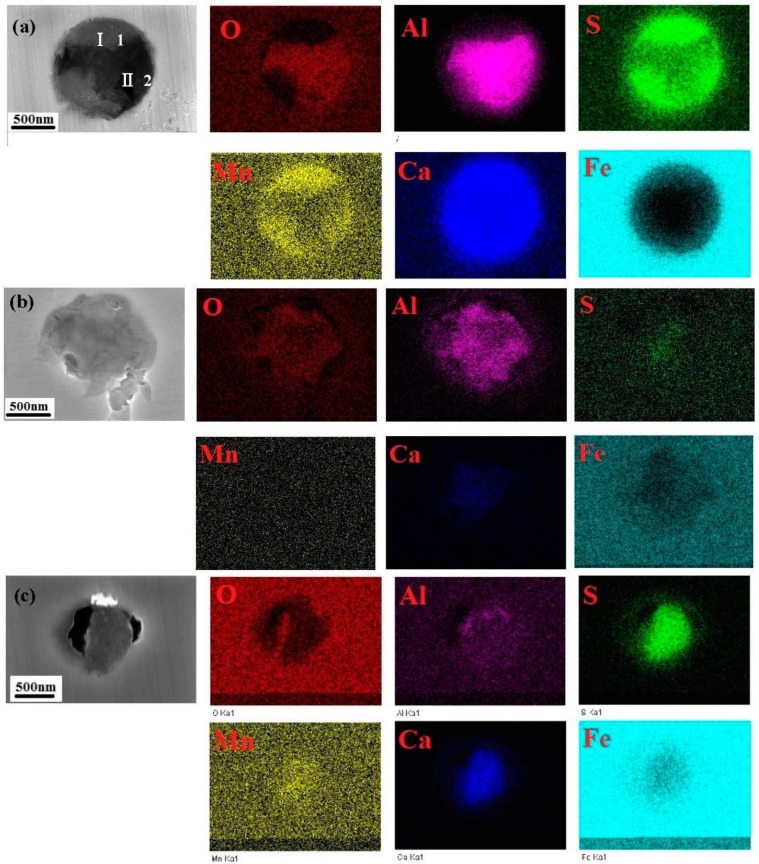
EDS composition maps of the inclusions in X70 steel: (**a**) Type A; (**b**) Type B and (**c**) Type C.

The elemental mapping analysis results of the inclusions in the X70 steel are shown in Figure 2. There are three inclusion types in the steel. Figure 2a shows that the type A inclusion consists of two parts; one part of the inclusion is rich in Mn, Ca and S, and the other part of the inclusion is rich in Al, Ca and O. Two part inclusions can be clearly distinguished from the morphology. This distribution of chemical composition and thermodynamic calculations [3,11] indicates that the complex inclusion is composed of the two different particles: (Al, Ca)O and (Mn, Ca)S. 

Figure 2b shows that the type B inclusion is rich in Al, Ca and O. In addition, small amounts of Mn and S elements are embedded in the inclusion. The type B inclusion is named the (Al, Ca)O inclusion. Figure 2c shows that the type C inclusion is rich in Mn, Ca and S. Small amounts of Al and O are embedded in the inclusion, and the type C inclusion is named the (Mn, Ca)S inclusion.

Table 3 shows the chemical composition of the inclusions shown in Figure 2. The three types of inclusions all contain Mn, S, Ca, Al and O, but the amounts of these elements in the inclusions are obviously different. Type B inclusions contain more oxides, while type C inclusions contain more sulphides.

Recent work shows that pitting corrosion is triggered by an unusual, high rate dissolution of inclusions [11,22]. Previous research focused on the effects of a single sulphide on stainless steel pitting corrosion. These reports propose that pitting has its genesis in the fabrication of the steel itself [23,24,25]. When the steel is poured, oxide particles form first, followed by the metal matrix solidification, while the sulphide particles form last [3,8]. As the sulphide cools, the composition of the inclusion itself (oxide, sulphide) and of the metal zone around it will change. In the case of the type A inclusion, when the steel is poured, the sulfide and oxide contents are more balanced. Although the composition of the two parts of the type A inclusion will change, they can be clearly distinguished from the morphology. For the type B inclusion, when the steel is poured, the oxide content is far higher than the sulphide, which can be embedded in the oxide inclusion where it cannot be clearly distinguished from the morphology of the two kinds of inclusions. In the case of the type C inclusion, when the steel is poured, the sulfide content is far higher than the oxide at the inclusions’ positions, and sulfide can enclose oxide inclusions completely.

### 3.3. Potentiodynamic Polarization Tests

**Figure 3:**
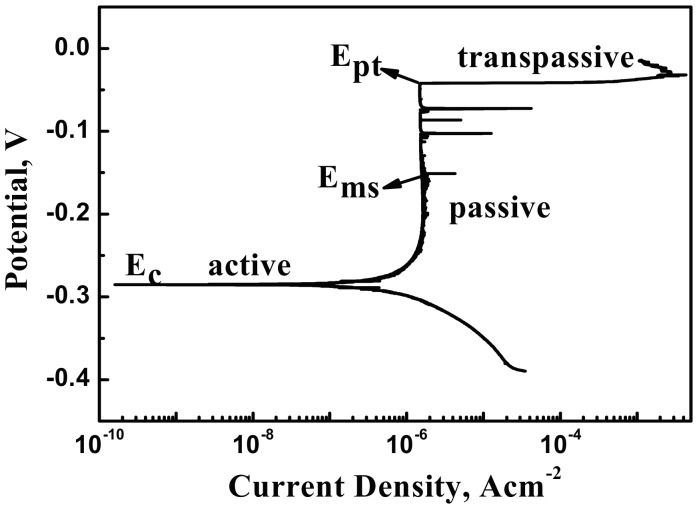
Potentiodynamic polarization test results of the X70 steel in 0.1 mol/L NaCl and 0.5 mol/L NaHCO_3_ neutral pH aqueous solutions.

Figure 3 shows the representative potentiodynamic polarization curve of the X70 steel in 0.1 mol/L NaCl + 0.5 mol/L NaHCO_3_ neutral pH solution. Active, passive and transpassive regions were identified in the potentiodynamic polarization curve. The appearance of current fluctuations in the passive region provides evidence that passivity is not stable, indicative of pitting corrosion [25]. This transience indicates active and repassivation processes of metastable pits. In the polarization curve, the potential corresponding to the first peak is referred to as the metastable pitting potential E_ms_ and the potential from which the current increases continually is denoted as the pitting potential E_pit_.

**Figure 4:**
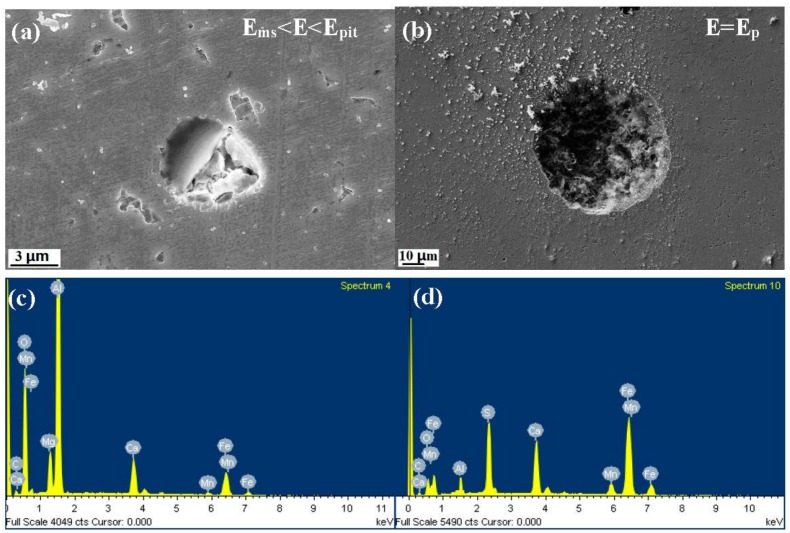
The morphology and EDS analysis of metastable pits at type A inclusion sites after the potentiodynamic polarization test. (**a**,**c**): E_ms_ < E < E_pit_; (**b**,**d**): E = E_pit_.

**Figure 5:**
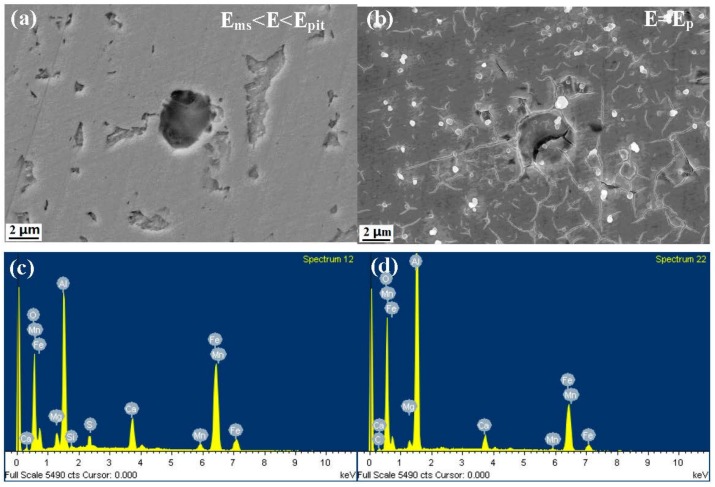
The morphology and EDS analysis of metastable pits at type B inclusion sites after the potentiodynamic polarization test. (**a**,**c**): E_ms_ < E < E_pit_; (**b**,**d**): E = E_pit_.

**Figure 6:**
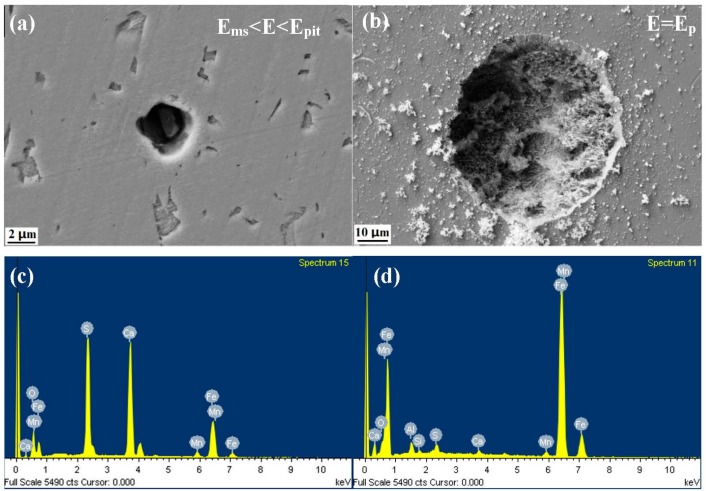
The morphology and EDS analysis of metastable pits at type C inclusion sites after the potentiodynamic polarization test. (**a**,**c**): E_ms_ < E < E_pit_; (**b**,**d**): E = E_pit_.

Figure 4, Figure 5 and Figure 6 show the morphology and EDS analysis of metastable pitting at inclusion sites after potentiodynamic polarization tests. When the polarization potential E is 30 mV above E_ms_ (E_ms_ < E < E_pit_), pitting was clearly generated at the inclusions’ positions. The passive film of the type A inclusion is broken at the site rich in Mn, Ca and S, as seen in Figure 4a,d. As seen in Figure 5a,c, the type B inclusion is rich in Al, Ca and O, and the passive film of the type B inclusion is intact. Figure 6a,c show that the type C inclusion is rich in Mn, Ca and S, the passive film of the type C inclusion is broken through and the inclusion is partially dissolved. The passive films of the type A and C inclusions are easier to break than the metal matrix surface.

With the increase of anodic potential, the inclusions were observed at the E = E_p_ potential. The type A inclusion had induced stable pitting (Figure 4b). The passive film of the type B inclusion began to rupture inside the inclusion and at the interface between the inclusion and the metal matrix (Figure 5b). The type C inclusions had induced stable pitting, where smaller inclusions were residues, as seen in Figure 6b,d.

According to the potentiodynamic polarization test results, the dissolution and passivation of inclusions are in competition. The inclusion dissolution rate is much slower than passivation. The inclusion is protected by passive film and not dissolved (Figure 4a). When the dissolution rate of the inclusion is much higher than the passivation, the inclusion will gradually dissolve, leading to the metal matrix being exposed to the corrosive solution (Figure 4c). The active process occurred. The metal matrix can still be passivated at this passive region potential. The repassivation process occurred.

Previous work found that metastable pits can be induced by inclusions. Meanwhile, Punckt et al. found that some metastable pits are active, but others are inactive. Active pits can remain metastable during the sharp rise in the corrosion rate [13]. The result shows that type A and C inclusions can lead to continuous metastable pitting corrosion. Type B inclusions cannot lead to metastable pitting corrosion generated continuously because stable passivation film is formed at the type B inclusion position. Thus, type A and C are active inclusions and type B is an inactive inclusion.

### 3.4. Characterization of the Corrosion Attack

The composition and structure of inclusions determine the mechanism of pitting initiation [26,27,28]. In order to verify the pitting process and pitting resistance of the three kinds of inclusions in the X70 steel, specimens’ surfaces after different immersion times were examined by SEM and EDS. The immersion test was conducted in the 0.1 mol/L NaCl + 0.5 mol/L NaHCO_3_ aqueous solution.

**Figure 7:**
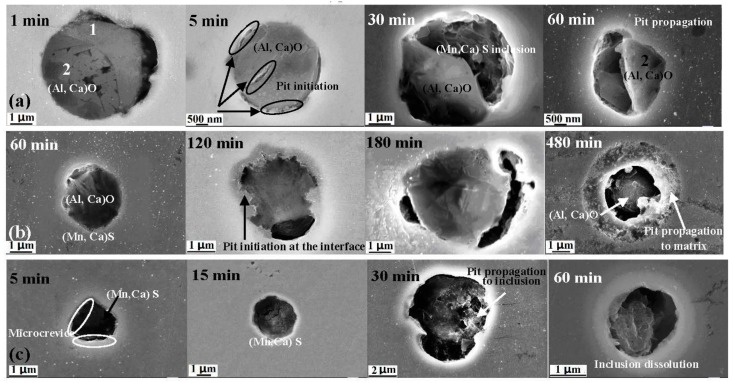
SEM images of three types of inclusions after initiation and propagation of pitting corrosion in X70 steel: (**a**) Type A; (**b**) Type B and (**c**) Type C.

Figure 7 shows images of the three types of inclusions in the X70 steel after immersion corrosion tests in 0.1 mol/L NaCl and 0.5 mol/L NaHCO_3_ solutions at 25 °C. As seen in Figure 7a for the type A inclusion, pitting corrosion was first initiated at the interface between the metal matrix and the (Mn, Ca)S inclusion part when the immersion time was 1 min. When the immersion time was prolonged to 5 min, pitting corrosion of the type A inclusion did not propagate from the interface into the matrix, but propagated to the (Mn, Ca)S inclusion. When the immersion time was prolonged to 60 min, pitting corrosion began in the matrix, but did not propagate to the (Al, Ca)O inclusion. Therefore, this indicated that in the X70 steel, the resistance to pitting corrosion for inclusions rich in Mn, Ca and S was lower than the metallic matrix and the type A inclusion was lower than the metallic matrix.

However, for the type B inclusion, pitting did not initiate when the immersion time was 60 min, as seen in Figure 7b. Pitting corrosion began to initiate at the interface between the metal matrix and the (Al, Ca)O inclusion when the immersion time was 120 min. The pitting initiation time of the type B inclusion was much longer than the type A inclusion. When the immersion time was prolonged to 180 min, the pitting corrosion of the X70 steel did not propagate to the stable (Al, Ca)O inclusion, but propagated to the metal matrix. The corrosion of the (Al, Ca)O inclusion and the metal matrix became more serious. When the immersion time was prolonged to 480 min, the residual inclusion was in the metal matrix, and pits propagated further into the metal matrix. This indicated that the resistance to pitting corrosion of the type B inclusion was higher than the metal matrix.

For the type C inclusion, pitting corrosion had initiated at the interface area between the metal matrix and (Mn, Ca)S inclusion when the immersion time was 1 min. When the immersion time was prolonged to 5 min, pitting corrosion of the X70 steel did not propagate from the interface area into the matrix, but propagated to the sulfide inclusions. When the immersion time was prolonged to 30 min, the metal matrix was exposed in solution when the manganese sulfide inclusions were dissolved further. Therefore, this indicated that in the X70 steel the resistance to pitting corrosion for inclusions, such as (Mn, Ca)S, was lower than the metal matrix.

Table 4 shows the element percentages of the three inclusion types after immersion for different times. For the type A inclusion, Mn, Ca and S percentages of the rich (Mn, Ca)S part were greatly reduced, while the element content of the (Al, Ca)O part showed little change after immersion for 60 min. For the type B inclusion, the element content did not significantly change after immersion for 480 min. For the type C inclusion, Al and O percentages were slightly reduced, while Mn, Ca and S percentages were greatly reduced. These results indicate that Mn, Ca and S elements are easily dissolved. These results obtained from the corrosion attack tests indicate that the pitting corrosion resistance type A < type C < type B; (Mn, Ca)S inclusion < matrix < (Al, Ca)O inclusions.

### 3.5. The Inclusions’ Potential Obtained by SKPFM

The activity of the inclusions can be expressed by the potential [29]. To demonstrate corrosion tendency (relative nobility) of the three kinds of inclusions present in the X70 steel, SKPFM measurements were performed to map Volta potential variations on the polished sample surface.

**Figure 8:**
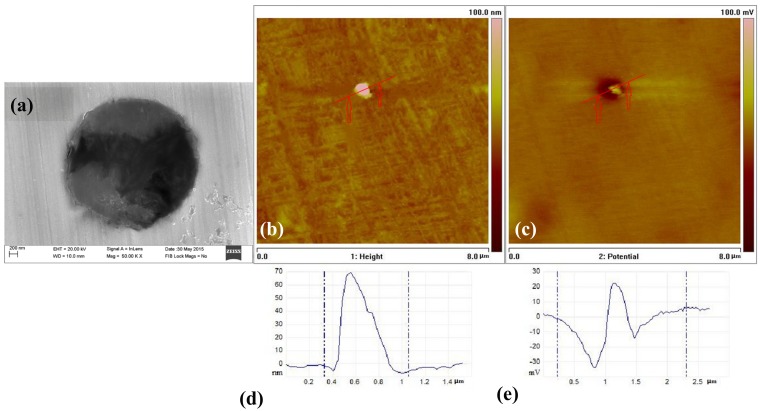
SEM and SKPFM images around the type A inclusion: (**a**) SEM image; (**b**) AFM topography image; (**c**) Surface potential distribution of the same area; (**d**) Topography section analysis and (**e**) Section analysis of the surface potential.

AFM morphology and Volta potential images of the type A inclusions are shown in Figure 8. The height variation in the surface was less than 2 nm with a spike and a little scratch in Figure 8d. Thus, the surface of the specimen can be considered flat. The higher (brighter) surface of the area was of the type A inclusion with a maximum height of 66 nm (Figure 8d). The inclusion slightly protruded after polishing because of the difference in hardness. On the other hand, variations in the Volta potential are clearly seen in Figure 8c. Both lower and higher potentials were observed around the inclusion. Low Volta potential indicates low relative corrosion resistance, while higher Volta potential indicates higher relative corrosion resistance. It is clearly seen that the (Al, Ca)O inclusion part had higher potential and the (Mn, Ca)S part had lower potential.

These results, obtained from the SKPFM Volta potential images, indicate that the (Al, Ca)O inclusions have a lower corrosion tendency compared with the matrix and the (Mn, Ca)S inclusions have a higher corrosion tendency compared with the matrix. These results are in agreement with the corrosion tests (Figure 5a).

**Figure 9:**
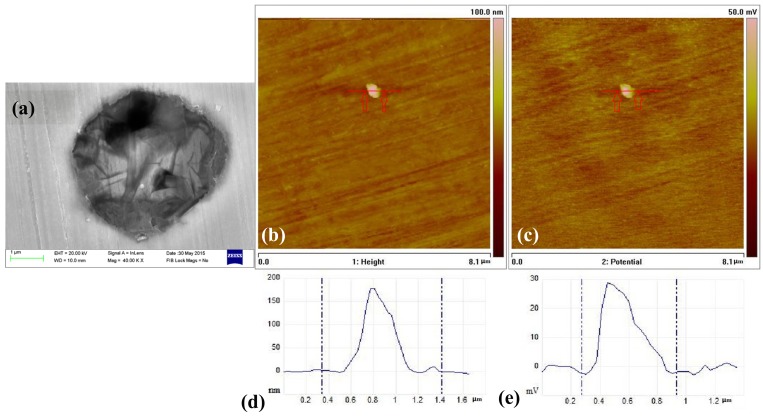
SEM and SKPFM images around the type B inclusion: (**a**) SEM image; (**b**) AFM topography image; (**c**) Surface potential distribution of the same area; (**d**) Topography section analysis; (**e**) Section analysis of the surface potential.

Figure 9 shows the AFM morphology and Volta potential images of the type B inclusions. The higher (brighter) surface of the area was of the type B inclusion with a maximum height of 175.7 nm (Figure 9b). On the other hand, higher potential was observed around the type B inclusion. This indicates that the type B inclusion has higher relative corrosion resistance than the metal matrix. A lower potential was observed around the interface between the inclusion and the matrix. This indicates that the interface has lower corrosion resistance than the metal matrix. Figure 6b shows that the pitting corrosion began to initiate at the interface between the metal matrix and the inclusion. Suter and Böhni [30,31] demonstrated via microelectrochemical capillary investigations that the electrochemical reactivity was highest at the inclusion and metal interface, and not in the inclusion and the matrix itself. The arrangement of the iron atoms is disordered at the interface between the inclusion and the metal matrix. The metal surface near the inclusion is rougher than the surface of the metal matrix. Thus, metal near the inclusion has higher energy than the metal matrix. The corrosion resistance near the inclusion is much worse than the metal matrix [5,31]. It is consistent with the interface having lower potential than the inclusion and the metal matrix, as seen in Figure 4. The corrosion test results and the AFM potential test results are also consistent.

**Figure 10:**
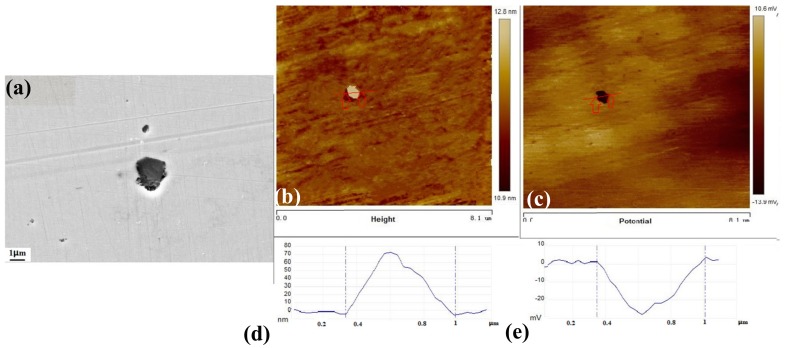
SEM and SKPFM images around the type C inclusion: (**a**) SEM image; (**b**) AFM topography image; (**c**) Surface potential distribution of the same area; (**d**) Topography section analysis; (**e**) Section analysis of the surface potential.

Figure 10 shows the AFM morphology and Volta potential images of the type C inclusions. The higher (brighter) surface of the area was of the type C inclusion with a maximum height of 71.7 nm (Figure 10b). On the other hand, lower potential was observed around the type C inclusion. This indicates that the type C inclusion has lower relative corrosion resistance than the metal matrix. This result is in agreement with the corrosion test (Figure 7c).

### 3.6. Pitting Initiation Mechanism

Figure 2 shows that there are three inclusion types in the X70 pipeline steel. Their pitting corrosion resistance is very different, according to the corrosion test results in Figure 7: type A < type C < type B. AFM results indicate that there is a potential difference between the inclusion and the metal matrix. The (Mn, Ca)S inclusion potential is higher than the matrix, while the (Al, Ca)O inclusion potential is lower than the matrix. Galvanic corrosion occurs at inclusions sites. Researchers have reported that inclusions induce galvanic corrosion in stainless steel [3,6].

**Figure 11:**
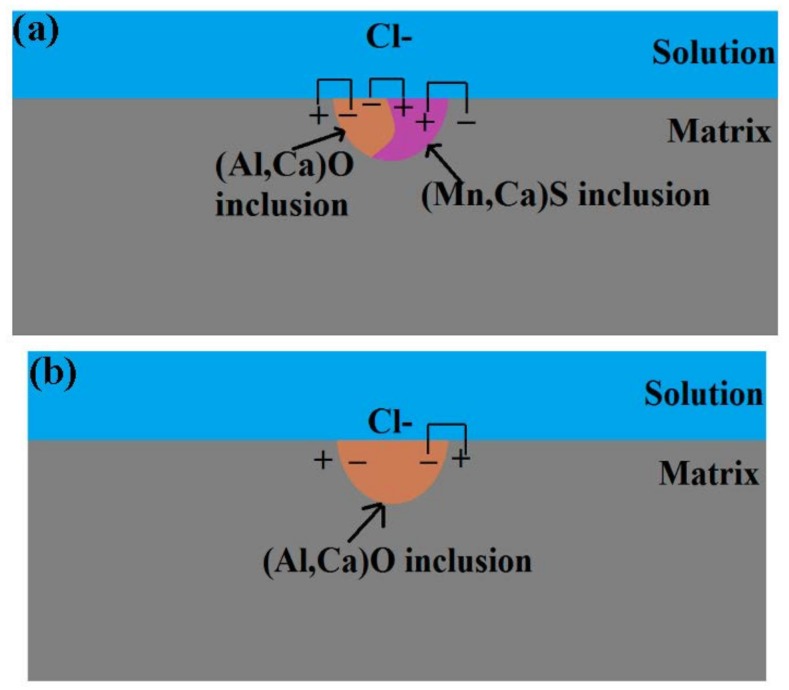

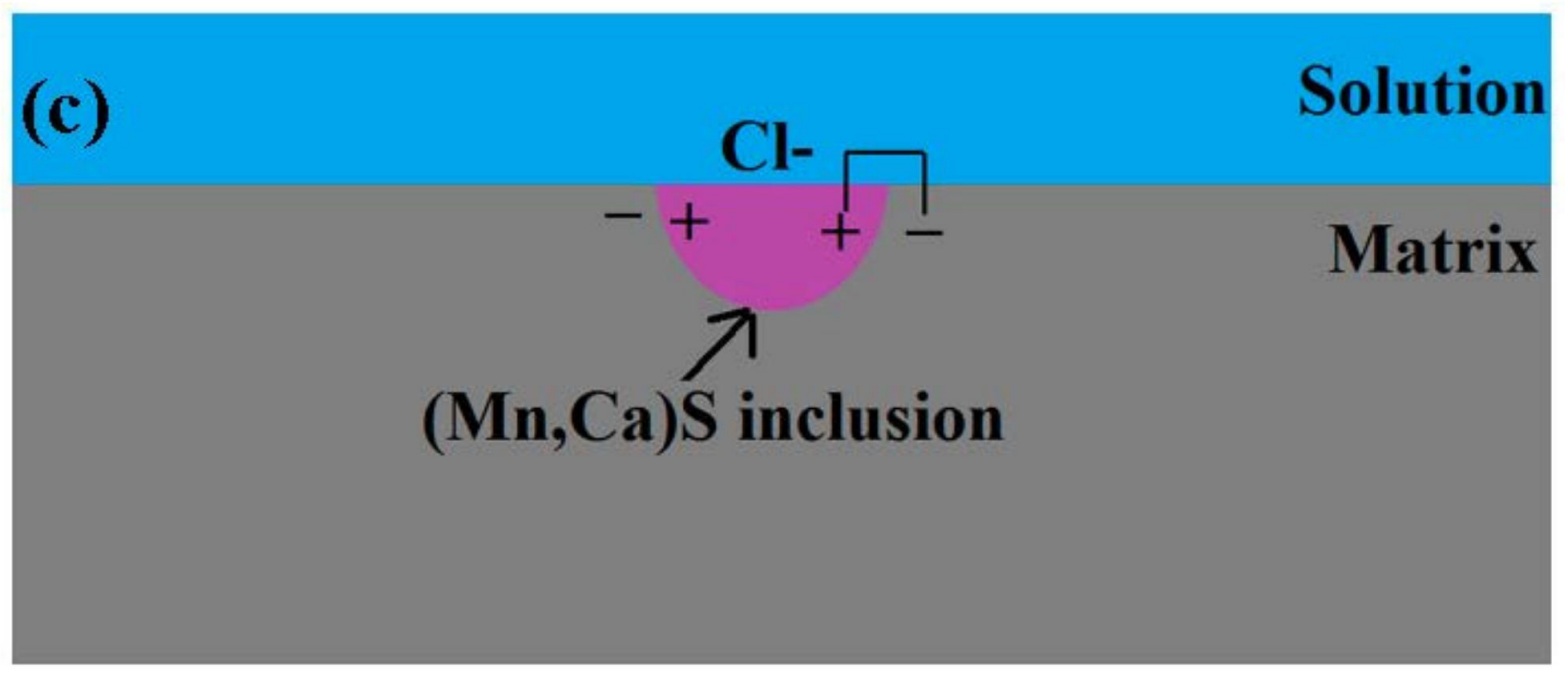
Schematic diagram of the inclusions’ effects on pitting corrosion initiation in X70 steel: (**a**) Type A; (**b**) Type B and (**c**) Type C.

According to the above results and analysis, the mechanisms for the initiation and propagation of pitting corrosion in the X70 steel are schematically presented in Figure 11. For the type A inclusion, the inclusion potential is as follows: (Mn, Ca)S inclusion < matrix < (Al, Ca)O inclusion, with the three galvanic cells in the type A inclusion area. The potential difference is the largest between the (Mn, Ca)S and (Al, Ca)O inclusions. Thus, the first (Al, Ca)O part is the cathode and the (Mn, Ca)S part is the anode. Pitting would initiate in 30 min at the (Mn, Ca)S inclusion part, as shown in Figure 7a. When the (Mn, Ca)S inclusions were dissolved, another galvanic corrosion occurred, where the (Al, Ca)O part is the cathode and the matrix is the anode. Pitting corrosion began to propagate to the matrix, as seen in Figure 7a. The pitting corrosion process includes pit initiation and propagation at the (Mn, Ca)S inclusion, followed by pit propagation to the interface between the (Al, Ca)O inclusion and the matrix, and pit propagation to the matrix, forming a stable pit.

Figure 7 shows that higher potential was observed around the type B inclusion and a lower potential was observed around the interface between the inclusion and the matrix. The schematic diagram includes only one galvanic cell in the type B inclusion area, where the inclusion is the cathode and the metal matrix is the anode. The pitting corrosion process includes pit initiation at the interface between the (Al, Ca)O inclusions and the matrix, followed by pit propagation to the matrix, forming a stable pit.

In the case of the type C inclusion, the pitting corrosion process includes pit initiation at the interface between the (Mn, Ca)S inclusion and the matrix, followed by pit propagation to the (Mn, Ca)S inclusion and the matrix, forming a stable pit.

## 4. Conclusions

Inclusion-induced pitting initiation mechanisms were investigated using SEM, SKPFM, immersion and electrochemical polarization tests. The results are summarized as follows:

(1) Three types of inclusions were observed in the X70 steel. Type A is a complex inclusion, one part is rich in (Mn, Ca)S and another part is rich in (Al, Ca)O. Type B is rich in (Al, Ca)O and has smaller amounts of (Mn, Ca)S. Type C is rich in (Mn, Ca)S and has smaller amounts of (Al, Ca)O.
(2) Three types of inclusions can induce pitting corrosion, but the pitting corrosion resistance is different: type A inclusion < type C inclusion < type B inclusion, and (Mn, Ca)S < matrix < (Al, Ca)O. The type A inclusion exhibited both lower and higher potentials than the matrix, while the type B inclusion exhibited higher potential than the matrix. Corrosion and AFM potential test results are consistent. Pitting corrosion is more likely to occur at the interface between the inclusion and the matrix because of its lower surface potential.
(3) The inclusions’ morphology was observed at different potentials. The passive film of the type A inclusion was broken at the potential E_ms_ < E < E_p_, while the type A inclusion can induce metastable pitting continuously. The passive film of the type B inclusion is inactive at the potential E_ms_ < E < E_p_, while the type B inclusion cannot induce metastable pitting continuously. The passive film of the type C inclusion was broken at the potential E_ms_ <E < E_p_, while the type C inclusion can lead to continuous metastable pitting corrosion. Thus, the types A and C are active inclusions and the type B is an inactive inclusion.
(4) Three kinds of possible mechanisms of inclusions inducing pitting corrosion were established for the X70 steel.

## Figures and Tables

**Table 1:** Chemical composition of the X70 steel specimens (wt %).

C	Si	Mn	P	S	Cr	Ni	Ca	Cu	Al	Ti	Other	Fe
0.066	0.29	1.39	0.008	0.002	0.032	0.201	0.051	0.17	0.034	0.015	0.300	Bal.

**Table 2:** Element percentages of the inclusions in Figure 1, obtained by EDS.

Type of Inclusion	Chemical Composition (wt %)					
	O	Al	Mn	S	Ca	Fe
b in Figure 1b	33.9	31.1	8.1	4.5	3.1	21.4
c in Figure 1c	31.8	36.7	0.6	0.5	0.4	30.1

**Table 3:** Element percentages of the inclusions in Figure 2, obtained by EDS.

Type of Inclusion	Chemical Composition (wt %)					
	O	Al	Mn	S	Ca	Fe
Type A in Figure 2a-1	28.62	8.44	12.01	11.48	8.64	30.80
Type A in Figure 2a-2	40.38	30.32	0.56	0.48	2.55	22.46
Type B in Figure 2b	29.8	18.59	1.88	4.46	4.88	37.96
Type C in Figure 2c	14.73	4.7	13.59	16.35	10.97	41.05

**Table 4:** Element percentages of inclusions in Figure 7, obtained by EDS.

Type of Inclusion	Chemical composition (wt %)					
	O	Al	Mn	S	Ca	Fe
Figure 7-a1-1 min	18.6	6.4	15.0	20.6	8.6	30.8
Figure 7-a1-60 min	0.2	0.3	1.6	0.2	0.2	97.5
Figure 7-a2-1 min	42.4	31.3	0.7	0.8	2.4	22.4
Figure 7-a2-60 min	40.4	31.4	0.6	0.5	1.5	25.6
Figure 7-b-60 min	29.8	18.6	1.9	4.5	4.8	37.9
Figure 7-b-480 min	31.8	20.9	0.9	1.5	0.8	44.1
Figure 7-c-5 min	11.6	4.5	16.6	19.4	11.9	36.0
Figure 7-c-60 min	5.7	1.7	1.6	2.4	1.9	86.7
